# Cases of Poisoning by Cyanide of Potassium and Cyanide of Silver

**Published:** 1851-09

**Authors:** 


*Cases of Poisoning by Cyanide of Potassium and Cyanide of Silver.*—It appears that, within a few years past, a liquid of a very poisonous nature, composed of cyanide of potassium and cyanide of silver, dissolved in distilled water, is sold in London, chiefly for the use of utterers of base coin. Cases of fatal poisoning from this article have been from time to time reported, and Mr. Letherby, in the London Medical Times,

12th July, 1851, gives the details of two additional cases. He states that "most of the cases of poisoning by this liquid have occurred among those who are engaged in the manufacture of base coin, and it is much to be regretted that a poison of so deadly a character should be accessible to a class of persons who are not over scrupulous in their dealings, or even cautious in their habits. It is sold to these persons at the charge of fourpence an ounce; this quantity is sufficient to kill four individuals, and conseqently, if murder were contemplated, it might be done at the rate of one penny per head.

The symptoms produced by the liquid are a little different from those which arise from the action of hydrocyanic acid, from pure cyanide of potassium, or even from cyanide of silver; for, in the first place. it does not commonly produce vomiting, and, in the second place, it does not generally cause convulsions, but, on the contrary, it occasions paralysis, with a perfect prostration of all the vital powers, and finally, death by coma. On making a post-mortem examination of the body, we find that the lungs are highly congested, that the bronchial tubes and pulmonic cells are filled with a frothy mucus, and that while the right side of the heart is gorged with black fluid blood, the left is empty, showing that the arrest of the circulation took place at the lungs.

